# Hybrid two-stage repair of Stanford A dissection with visceral or peripheral malperfusion

**DOI:** 10.1186/s13019-020-01307-8

**Published:** 2020-09-24

**Authors:** Zanxin Wang, Xianmian Zhuang, Bailang Chen, Junmin Wen, Minxin Wei

**Affiliations:** 1 Department of Cardiac Surgery, Fuwai Hospital Chinese Academy of Medical Sciences, Shenzhen, Guangdong People’s Republic of China; 2 Department of Cardiac Surgery, The University of Hong Kong – Shenzhen Hospital, Shenzhen, Guangdong People’s Republic of China; 3 Department of Intensive Care, Fuwai Hospital Chinese Academy of Medical Sciences, Shenzhen, Guangdong People’s Republic of China

**Keywords:** Aortic dissection, Malperfusion, Hybrid procedure, Thoracic endovascular aortic repair

## Abstract

**Background:**

The present study aimed to evaluate the effect of two-stage hybrid aortic repair at the distal aorta of Stanford A dissection with malperfusion.

**Methods:**

This retrospective case series included 20 patients with Stanford A dissection administered two-stage thoracic endovascular aortic repair (TEVAR) about 1 month after central repair because of visceral or limb malperfusion. The patients were examined by computed tomography (CT) angiography at 3, 6, 12 and 24 months after operation. Recovery of malperfusion and true lumen index were evaluated during follow-up.

**Results:**

Twenty patients underwent two-stage hybrid aortic repair, including 11 males and 9 females. The follow-up time was 24 ± 7 months. No intervention-related complications were observed, including stent graft-induced new re-entry tears, death, stroke and spinal cord injury. Malperfusion in all cases was corrected. The true lumen was not enlarged enough 1 month after the first surgery. Thrombosis of the false lumen was observed around the elephant trunk at the carina level and the celiac artery. Three months after second stage TEVAR, the false lumen thrombosis was resorbed; in addition, the trunk was fully expanded at the carina level, and the true lumen was enlarged at the celiac artery.

**Conclusions:**

Two-stage hybrid aortic repair for residual true lumen in the distal aorta 1 month after initial surgery is helpful for descending aorta remodeling and effective in treating malperfusion. This procedure may be a good option for patients suffering from Stanford A dissection with small true lumen in the distal aorta and malperfusion.

## Background

Stanford A aortic dissection is an acute condition characterized by the disruption of the thoracic aortic media layer caused by intramural bleeding separating the aortic wall layers; it leads to the formation of true and false lumen with or without communication [1–4]. Open surgery (total arch replacement combined with elephant trunk stent implantation) is routinely applied in most cardiac centers. However, it is only the first step aiming to avoid lethal complications [5]. Small true lumen in distal descending aorta leading to malperfusion is a potential risk factor for poor long-term prognosis [6].

The malperfusion syndrome can affect different vascular beds depending upon the exact location of the aortic tear: spinal cord, visceral, renal, and lower extremities [7, 8]. It is found in 20–40% of patients with Stanford A aortic dissection. Nearly 20% of these individuals require additional revascularization [9, 10]. The malperfusion syndrome may be complicated by a wide variety of symptoms. Nevertheless, early diagnosis is important in avoiding damage to organs and the subsequent inflammatory cascade that can affect the treatment success and patient prognosis [7, 8].

The hybrid procedure uses thoracic endovascular aortic repair (TEVAR) of distal descending aorta. Type III hybrid aortic repair, which encompasses total arch replacement with the descending elephant trunk, combined with second stage TEVAR, is indicated for acute aortic dissection with malperfusion or rupture [11–17]. Studies on second stage repair of distal residual aorta have prompted surgeons to seek the optimal time and location for performing TEVAR and avoiding complications. Therefore, the present retrospective study aimed to describe our experience and evaluate the effectiveness of Type III hybrid aortic repair at the distal aorta of acute Stanford type A aortic dissection with malperfusion.

## Methods

### Patients

This was a retrospective study. A total of 132 patients with Stanford A aortic dissection from January 2015 to December 2017 were treated in Fuwai Hospital Chinese Academy of Medical Sciences Shenzhen and Tianjin Medical University General hospital. Twenty of them underwent second stage TEVAR because of limb or visceral malperfusion, diagnosed both by clinical symptoms and radiographic findings.

This study was approved by the ethics committees of Fuwai Hospital Chinese Academy of Medical Sciences Shenzhen and Tianjin Medical University General Hospital. The need for individual consent was waived by the committees because of the retrospective nature of the study.

Malperfusion was reflected by symptoms or signs of compromised blood flow to a limb or visceral vessels. To diagnose limb malperfusion, pulse deficit was necessary, and computed tomography (CT) angiography was used to corroborate this clinical findings. Visceral malperfusion was determined via a combination of clinical and radiographic factors, including radiographic evidence of flow obstruction with clinical indicators of ischemia to the abdominal viscera such as melena or abdominal pain with distention.

### Surgical procedures

The diagnosis of acute Stanford A dissection was made by CT angiography [1].

The initial repair of acute Stanford A dissection was open surgery by the frozen elephant trunk (FET) technique: total arch replacement combined with elephant trunk stent implantation. All patients underwent CT scan within 3 months after the initial repair depending on the recovery of the distal aorta and malperfusion. Those still with malperfusion received TEVAR using a standardized protocol [18–21].

TEVAR was deployed using a graft landing zone of at least 2 cm via the retrograde femoral approach. The stent-graft diameter exceeded the diameter of the landing zone by at least 10–15%. All TEVAR procedures were performed under local anesthesia with invasive blood pressure monitoring.

### Follow-up

The patients were followed up at 3, 6, and 12 months, and then annually at the outpatient department. Aortic CT angiography was performed at each follow-up.

Total aortic diameter (adventitia to adventitia), true lumen diameter, and false lumen diameter were measured on CT scans at the celiac artery. The true lumen index was the true lumen diameter relative to total aortic diameter, and was calculated to evaluate the impact of second stage TEVAR on distal aorta remodeling [16].

### Statistical analysis

All statistical analyses were performed with SPSS 25.0 (IBM, Armonk, NY, USA). Continuous variables are mean ± standard deviation (SD), and were compared by the Student’s t test. Categorical variables were displayed as frequency (%) and analyzed by the chi-square test or fisher’s exact test. Repeated measures analysis of variance was performed for intragroup comparisons at different time points. Two-sided *P* < 0.05 was considered statistically significant.

## Results

**Fig. 1:**
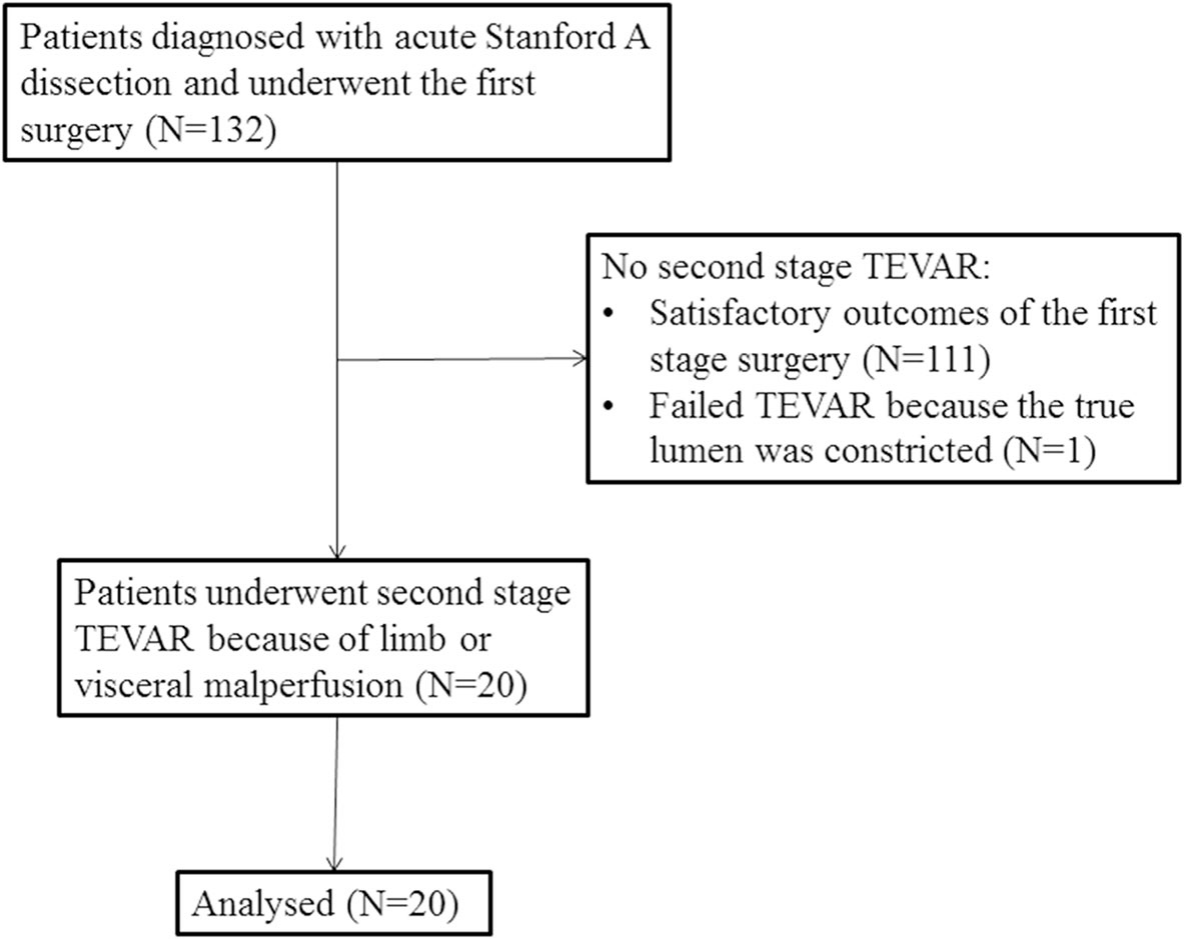
A total of 132 patients with Stanford A aortic dissection underwent an open surgery. Twenty of them underwent second stage TEVAR because of limb or visceral malperfusion and were enrolled

Twenty patients underwent second stage TEVAR (Fig. 1), including 11 males and 9 females. The average age was 45.1 ± 12.2 years. Baseline and surgical information is shown in Table 1. The reasons for TEVAR are listed in Table 2. There were no death, stroke, or spinal cord injury cases during or after TEVAR.

**Table 1:** Baseline characteristics

Characteristic	Two-stage hybrid aortic repair (*n* = 20)
Age (years)	45.1 ± 12.2
Gender, n (%)	
Male	11 (55.0)
Female	9 (45.0)
BMI (kg/m^2^)	26.2 ± 4.8
Hypertension, n (%)	16 (80.0)
Smoking, n (%)	12 (60.0)
Total bypass time (min)	212.3 ± 57.1
Arrest time (min)	26.2 ± 18.7
Operative time (min)	493.6 ± 79.2
Ventilation time (h)	102.6 ± 39.3

*BMI* Body mass index

**Table 2:** TEVAR information of the patients

No.	Gender	Age (years)	Reason for TEVAR	Stent in TEVAR	Surgery interval
1	Male	35	Narrow true lumen, limbs malperfusion	Microport 2,624,160	1 month
2	Female	42	Narrow true lumen, limbs malperfusion	Microport 2,826,160	1 month
3	Male	33	Narrow true lumen, limbs malperfusion	Microport 2,624,160	1 month
4	Male	40	Narrow true lumen, visceral malperfusion	Relay 2,826,170	1 month
5	Male	38	Narrow true lumen, visceral malperfusion	Microport 2,624,160	1 month
6	Male	60	Raising distal part of the stented elephant trunk, limbs malperfusion	Microport 3,026,160	2 weeks
7	Male	51	Narrow true lumen, limbs malperfusion	Microport 2,624,160	1 month
8	Female	45	Narrow true lumen, visceral malperfusion	Microport 2,624,160	1 month
9	Female	38	Narrow true lumen, visceral malperfusion	Microport 2,624,160	1 month
10	Female	42	Narrow true lumen, visceral malperfusion	Microport 2,826,160	1 month
11	Female	45	Narrow true lumen, limbs malperfusion	Microport 2,624,160	1 month
12	Female	31	Narrow true lumen, limbs malperfusion	Microport 2,826,160	3 weeks
13	Female	28	Narrow true lumen, limbs malperfusion	Microport 2,624,160	3 weeks
14	Male	31	Endoleak at distal part of the stent, visceral malperfusion	Microport 2,826,160	1 month
15	Male	48	Narrow true lumen, visceral malperfusion	Microport 2,826,160	1 month
16	Male	43	Narrow true lumen, visceral malperfusion	Microport 2,826,160	3 weeks
17	Male	71	Narrow true lumen, visceral malperfusion	Microport 2,826,160	3 weeks
18	Male	53	Narrow true lumen, visceral malperfusion	Microport 2,826,160	1 month
19	Female	62	Narrow true lumen, visceral malperfusion	Microport 2,624,160	1 month
20	Female	66	Narrow true lumen, visceral malperfusion	Microport 2,826,160	1 month

**Fig. 2:**
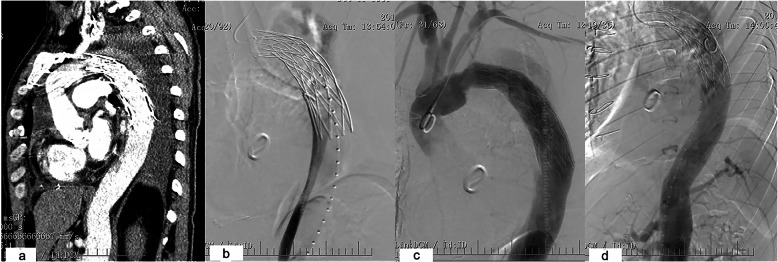
**a** The elephant trunk was found in the wrong place based on CT. **b** In the angiography of the femoral artery, the true lumen was constricted and very small. **c** Trunk in the false lumen. **d** The blood supply of abdominal organs was from the false lumen

One patient had failed TEVAR, because the elephant trunk was inserted into the false lumen. Aortic angiography showed the true lumen was constricted and very small. Blood flow in main vessels of the abdominal organs was normal. The descending aorta was not enlarged, and the patient had no complaint (Fig. 2). Follow-up is still going on. Further intervention will be performed later. His data are not shown in Table 2.

**Fig. 3:**
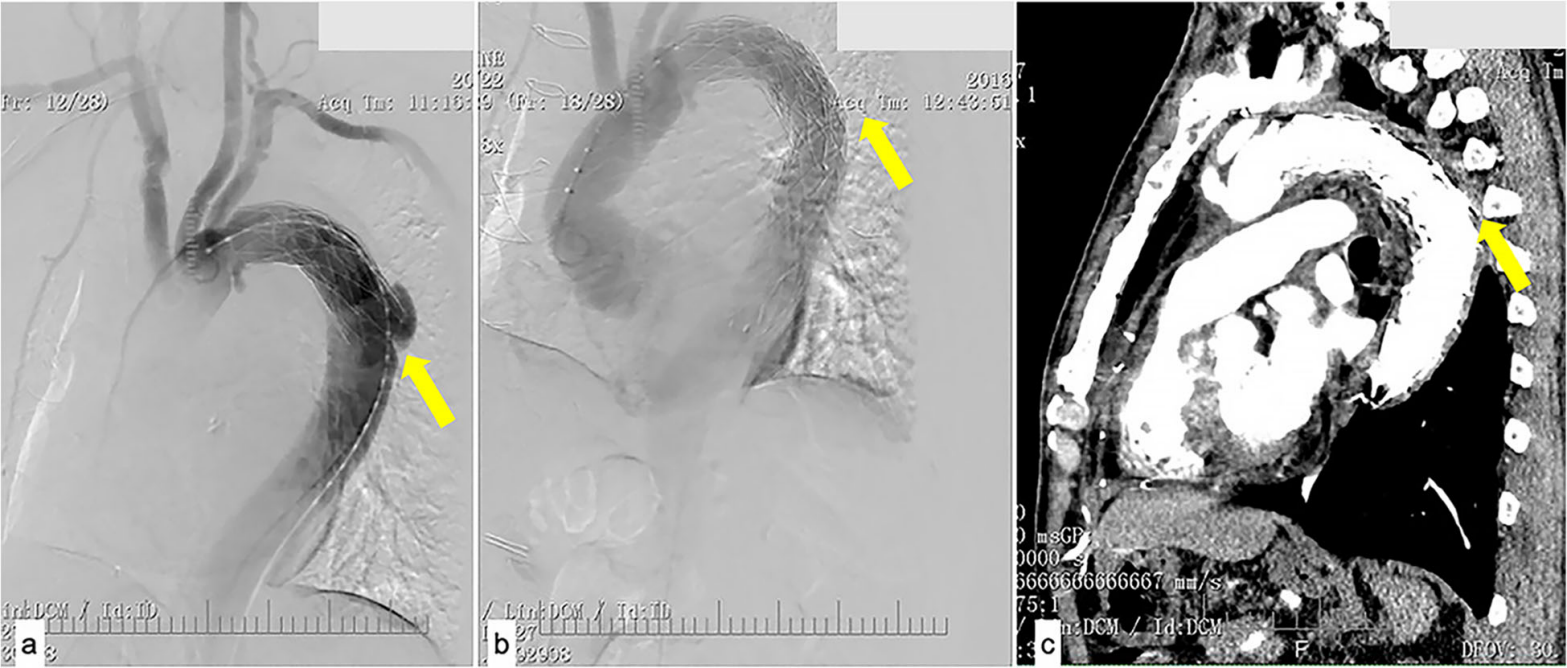
**a** Angiography illustrating that the distal part of the elephant trunk was raised to an ulcer of the descending aorta. **b** Second-stage TEVAR covered the ulcer. **c** At one-month follow-up, aorta remodeling was efficient

In another case, the distal part of the elephant trunk was inserted into an ulcer of the descending aorta in the first surgery (Fig. 3a). This was found by CT angiography 2 weeks after the operation. With limb malperfusion, second stage TEVAR was performed. The ulcer was covered, and the malperfusion was resolved (Fig. 3b, c).

**Fig. 4:**
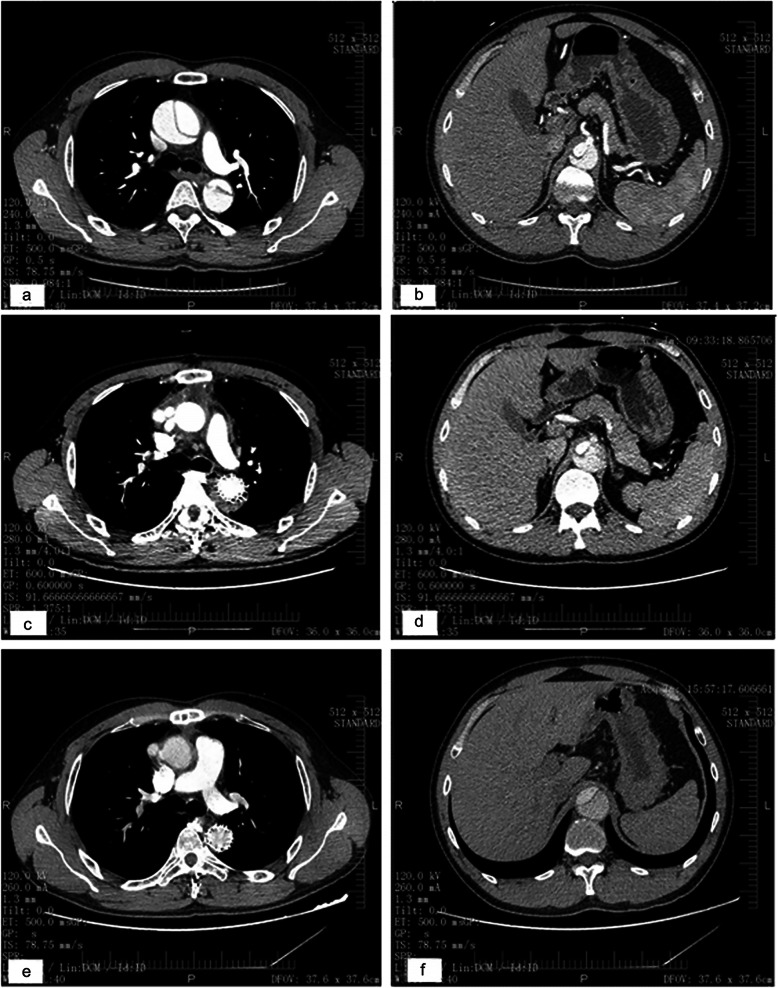
After Stanford A dissection, the true lumen was compressed by a huge false lumen before surgery at the carina (**a**) and celiac artery (**b**) levels. Thrombosis of the false lumen was observed around the stented graft at the carina (**c**) and celiac artery (**d**) levels 1 month after the first operation. TEVAR was performed and 3 months later, false lumen thrombosis was resorbed; in addition, the elephant trunk was fully expanded at the carina level (**e**), and the true lumen was enlarged at the celiac artery level (**f**)

All patients underwent computed tomography. The true lumen was compressed by a huge false lumen before surgery at the carina (Fig. 4a) and celiac artery (Fig. 4b) levels. Thrombosis of the false lumen was observed around the elephant trunk at the carina and celiac artery levels 1 month after the first stage surgery (Fig. 4v). Three months after TEVAR, false lumen thrombosis was resorbed; in addition, the trunk was fully expanded at the carina level, and the true lumen was enlarged at the celiac artery level (Fig. 4e, f).

The aortic data at the celiac artery level are displayed in Table 3. The true lumen was not enlarged enough after the first surgery. After TEVAR, substantial improvement was noted. The true lumen index increased to 56.8 ± 10.9% (*P* < 0.05 compared to the value after the first surgery).

**Table 3:** Aortic data at the celiac artery level

Variable	Before surgery	1 month after the first surgery	3 months after TEVAR	6 months after TEVAR	12 months after TEVAR	24 months after TEVAR
True lumen (mm)	10.2 ± 2.4	12.2 ± 2.9^a^	17.6 ± 4.5^ab^	19.1 ± 3.6^abc^	21.2 ± 4.4^abc^	22.1 ± 5.8^abc^
False lumen (mm)	17.8 ± 2.9	16.1 ± 3.5^a^	13.6 ± 4.1^ab^	11.2 ± 2.1^abc^	10.1 ± 3.5^abc^	9.4 ± 5.5^abc^
True lumen index (%)	37.1 ± 6.4	43.4 ± 9.1^a^	56.8 ± 10.9^ab^	63.1 ± 9.8^abc^	67.3 ± 10.1^abc^	69.1 ± 11.2^abc^

a. vs. preoperative, P < 0.05

b. vs. 1 months after first surgery, P < 0.05

c. vs. 3 months after re-intervention, P < 0.05

## Discussion

Hybrid techniques for the treatment of Stanford A dissection are increasingly applied. Type III hybrid procedure has its own advances in patients with one or more entry tears in distal descending aorta. It was reported that 20–40% of patients require TEVAR to the descending aorta for the repair of Stanford A aortic dissection because of a small true lumen in the distal aorta, with or without malperfusion [22]. In the present case series, 20 patients underwent TEVAR about 1 month after the first operation. The results showed that second stage TEVAR was effective for descending aorta remodeling, with increased true lumen index and decreased false lumen index. In addition, it was effective in treating malperfusion. Limbs and visceral perfusion recovered well after TEVAR.

We performed TEVAR in the second stage surgery in all patients. The thrombosis in the false lumen was resorbed, and the true lumen might be enlarged about 1 month after the central procedure. Meanwhile, the incidence of paraplegia may increase if the patient underwent central surgery and TEVAR simultaneously. Based on our clinical experience, a 1-month period is a good timing for TEVAR after the first surgery, with good thrombosis outcomes because the aortic intima is not yet stable. Waiting for a long time after the first surgery might lead to difficulties such as narrowing of the true lumen.

In addition, open surgery has some limitations [23]. First, the elephant trunk is not long enough for some patients. Secondly, in some complex cases, it could be inserted into the wrong place of the descending aorta, especially in case of first entry tears at the arch and descending part of the aorta. In one case in this series, the elephant trunk was inserted into the false lumen. In another patient, the distal part of the trunk was placed in the ulcer of the descending aorta. Compared with conventional repair, the hybrid approach could be safer for complex conditions, because of a wire from the femoral artery to the arch. This could ensure the surgeon inserts the stent into the true lumen by angiography. In addition, the hybrid surgery results in 80–100% of false lumen thrombus formation in proximal descending aorta (stented segment) [24–26].

The most common reasons for TEVAR in aortic dissection are endoleak, false lumen perfusion, and aortic dilatation [27, 28]. In the present case series, the indications for TEVAR were narrow true lumen, limb malperfusion, and visceral malperfusion. Additional studies are necessary to examine the risk factors for TEVAR after open repair of type A aortic dissection. The true lumen index represents the area of an aortic section providing blood to the distal organs, and could be considered a good indicator of organ and limb perfusion [29], indicating the need for TEVAR to increase this parameter. Indeed, a narrow true lumen and a low true lumen index are subsequent to false lumen perfusion, since elevated false lumen perfusion leads to reduced true lumen perfusion. As shown in the present study, the true lumen index was only marginally increased after the first surgery, but increased more significantly after TEVAR. At our center, we use the true lumen index to evaluate the status of distal aortic remodeling. However, the need for TEVAR is based on a comprehensive assessment of blood supply to the distal organs.

Nevertheless, TEVAR is not without risk, including spinal cord injury [18, 30], probably by covering the main vessels feeding the spinal cord. Therefore, the endografts used in second stage-TEVAR are shorter than those in Stanford B dissection. In the current cases, most endografts were 160 mm in length, and were placed above the celiac artery level to preserve blood flow to abdominal organs.

The present study had some limitations. It was a retrospective observational study with potential selection bias. The sample size was small for patients administered TEVAR, and follow-up was short. The study population was young, and it should be evaluated whether this approach would be valuable in older and elderly patients. Nevertheless, aortic dissection occurs at a younger age in China than in Western countries [30].

## Conclusions

In conclusion, two-stage TEVAR at the residual true lumen in the distal aorta after initial surgery for Stanford A aortic dissection is effective in distal aorta remodeling at about 1 month after the first operation. In addition, it is useful for treating malperfusion. The above results suggest that this approach has low operative morbidity and mortality. The hybrid two-stage repair may be a good option for patients suffering from Stanford A dissection with a small true lumen in the distal aorta and malperfusion. Simultaneously endografting the distal descending aorta could also be considered in complex cases. Further investigation of the safety and long-term efficacy of this method is warranted.

## Data Availability

The datasets analyzed in the current study are available from the corresponding author upon reasonable request.

## Abbreviations

TEVAR: Thoracic endovascular aortic repair
MRI: Magnetic resonance imaging
CPB: Cardiopulmonary bypass
SD: Standard deviation
